# EFFICIENT RIBOSOMAL RNA DEPLETION FROM *DROSOPHILA* TOTAL RNA FOR NEXT-GENERATION SEQUENCING APPLICATIONS

**DOI:** 10.12688/wellcomeopenres.23509.2

**Published:** 2025-05-13

**Authors:** Omkar Koppaka, Shweta Tandon, Ankita Chodankar, Awadhesh Pandit, Baskar Bakthavachalu

**Affiliations:** 1 School of Biosciences and Bioengineering, Indian Institute of Technology Mandi, Mandi, Himachal Pradesh, 175005, India; 2 National Centre for Biological Sciences, Tata Institute of Fundamental Research, Bangalore, Karnataka, 560065, India

**Keywords:** Drosophila, rRNA, RNase H, Next-Generation Sequencing

## Abstract

We developed a cost-effective enzyme-based rRNA-depletion method tailored for *Drosophila melanogaster*, addressing the limitations of existing commercial kits and the lack of peer-reviewed alternatives. Our method employs single-stranded DNA probes complementary to *Drosophila* rRNA, forming DNA-RNA hybrids. These hybrids are then degraded using the RNase H enzyme, effectively removing rRNA and enriching all non-ribosomal RNAs, including mRNA, lncRNA and small RNA. When compared to a commercial rRNA removal kit, our approach demonstrated superior rRNA removal efficiency and mapping percentage, confirming its effectiveness. Additionally, our method successfully enriched the non-coding transcriptome, making it a valuable tool for studying ncRNA in *Drosophila*. The probe sequences and rRNA-depletion protocol are made freely available, offering a reliable alternative for rRNA-depletion experiments.

## Plain language summary

*Drosophila melanogaster* is a model organism that plays an important role in understanding various conditions in health and disease. RNA helps in the maintenance of organismal health, while dysregulation leads to diseases. Various methods are available to study RNA expression at small- and large-scale levels. One of the most useful of these is the method called RNA sequencing. However, for good quality data, the rRNA, which is around 80% of total RNA, has to be removed. We use custom single-stranded DNA probes, which bind to the rRNA molecules. These rRNA:ssDNA complexes can then be removed using RNAse H, which specifically depletes RNA:DNA hybrids. Using this method, we were able to deplete ~97% of rRNA.

## Introduction

*Drosophila melanogaster* has emerged as a key model organism due to rapid generation time, easy maintenance, well-characterized genome and low experimental cost ( Mirzoyan *et al.*, 2019; Tolwinski, 2017). With a genome that shares ~60% homology with humans of which 75% are linked to human diseases ( Adams *et al.*, 2000; Banfi *et al.*, 1996; Fortini *et al.*, 2000), *Drosophila* plays a critical role in studying numerous conditions such as neurodegeneration, cardiac diseases, metabolic disorders and even rare human diseases ( Goodman & Bellen, 2022; Kim *et al.*, 2021; Link & Bellen, 2020; Souidi & Jagla, 2021). Beyond disease models, *Drosophila* is also essential for exploring normal physiology and gene regulation, as its transcriptome provides insights into conserved cellular mechanisms and gene interactions fundamental to development and cellular function ( Hardin *et al.*, 1990; Harvey *et al.*, 2003; Nüsslein-Volhard & Wieschaus, 1980). This model organism enables researchers to investigate gene regulation under a range of conditions, advancing our understanding of both healthy and pathological states.

RNA expression plays a crucial role in maintenance of physiological balance, as evidenced by the fact that dysregulation of RNA leads to several pathological conditions ( Bradley & Anczuków, 2023; Nemeth *et al.*, 2024). To understand the roles of RNA expression in physiological and pathological conditions, identifying both global and tissue-specific gene expression patterns is crucial. Earlier methods, such as Sanger sequencing, Northern blotting, and qRT-PCR, were both labor and time-intensive and could only analyze a small portion of the transcriptome. The introduction of high-throughput methods, such as RNA sequencing and its variants, have transformed transcriptomic research ( Daines *et al.*, 2011; Tang *et al.*, 2009; Wang *et al.*, 2009). RNA sequencing now allows for comprehensive profiling of gene expression, providing a broad and detailed view of transcriptomic dynamics. These advancements have significantly improved our capacity to study complex biological processes and diseases at a molecular level, advancing our understanding of various complex conditions.

**Figure 1.**
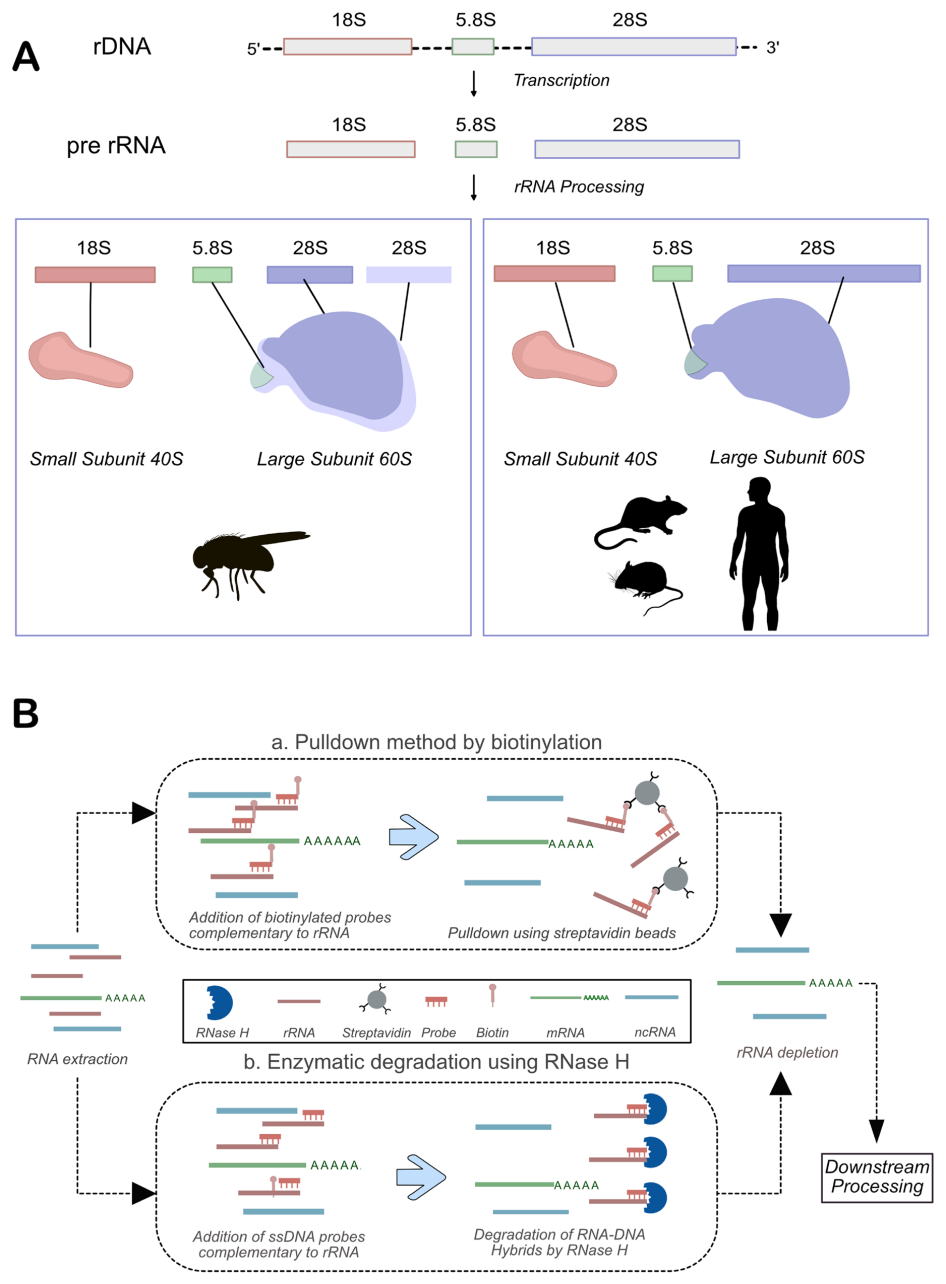
rRNA biogenesis and depletion methods. **A**) The rRNA biogenesis pathways differ in *Drosophila* and Mammals. While mammals have one fragment of 28S rRNA, Drosophila 28S rRNA has 2 fragments: α and ß. ( **B**) A schematic for using biotinylated probes or RNAse H for rRNA-depletion.

Ribosomal RNA (rRNA), the most abundant type of RNA, typically constitutes about 80% of total RNA ( Singer & Berg, 1991), posing a significant challenge for profiling the RNA types relevant for gene expression studies. Therefore, it must be removed from total RNA before sequencing, to allow for the enrichment of high-value targets ( Huang *et al.*, 2011; O’Neil *et al.*, 2013). Currently, two methods exist for rRNA removal: polyA-enrichment and rRNA-depletion. PolyA-enrichment is widely used because of its cost-effectiveness and ability to work effectively with low sequencing depth, often providing better coverage compared to rRNA depletion at equivalent sequencing depth. Unfortunately, polyA-enrichment sometimes leads to a 3’ end bias ( Tariq *et al.*, 2011) which renders it less effective for low-quality RNA or Formalin-Fixed Paraffin-Embedded (FFPE) tissue samples, which generally have degraded RNA ( Kellman *et al.*, 2021; Zhao *et al.*, 2014). While this means that rRNA that lack polyA tail are eliminated, it also leads to the loss of many non-coding RNA (ncRNA) lacking a polyA tail. rRNA-depletion, which focuses on the elimination of rRNA rather than the selection of a particular class of RNA serves as a much better alternative for low-quality and degraded RNA while also enriching the non-coding RNA that would otherwise be lost in the polyA-selection method ( Cui *et al.*, 2010; Kissopoulou *et al.*, 2013). Depletion of rRNA generally depends on the principle of hybridization of single-stranded rDNA probes to rRNA. Thus, for efficient rRNA depletion and minimal off-target activity, these probes need to be highly specific for the species in which rRNA depletion must be done. This restricts the use of the kits to the intended organisms and those that share their conserved rRNA regions ( Kraus *et al.*, 2019). rRNA sequences are relatively conserved across eukaryotes, especially higher eukaryotes. Hence, kits developed for humans also work for rats and mice. However, in the case of insects, the ribosome biogenesis pathways are different. The 28S rDNA has 2 types of insertions; while Type I insertion resembles mammalian rDNA with a single block for 28S rDNA, Type II insertion has 2 blocks of 28S rRNA that are separated from each other by 5.4 kb of DNA ( Glover & Hogness, 1977; Pellegrini *et al.*, 1977; Tautz *et al.*, 1988; White & Hogness, 1977). 28S rRNA is transcribed from these rDNA along with 5.8S and 18S as a single large precursor. Following splicing, the 28S rRNA undergoes further processing through an unknown mechanism which leads to fragmentation of the 28S rRNA into α and ß fragments ( Dawid & Wellauer, 1978; Kidd & Glover, 1981; Winnebeck *et al.*, 2010). Due to this fragmentation of the insect rRNA during processing, the commonly available kits used for vertebrate rRNA depletion, such as RiboZero and RiboZero Gold do not completely remove 5S rRNA or 28S rRNA, which leads to low mapping and sequence coverage. Thus, most commercially available kits do not recommend using kits developed for a particular organism or group of organisms to be used for rRNA depletion in other organisms ( Figure 1A). This inefficiency can negatively impact downstream analyses, such as sequence mapping and coverage. Due to the lack of an efficient protocol, several previous studies have performed rRNA depletion using commercial kits designed for vertebrates in *Drosophila* ( Table 1).

**Table 1.** rRNA-depletion kits previously used for *Drosophila* samples.

Name of Kit	Recommended Species	Notes	Reference
Tecan Ovation ^®^ RNA-Seq System V2	-	cDNA synthesis without polyA selection or rRNA depletion	( Hughes *et al.*, 2012)
Zymo-Seq RiboFree Total RNA library Kit	Any	20% of Insect rRNA remains undepleted	( Talross & Carlson, 2023)
Illumina Ribo-Zero™ Gold Kit	HMR	Currently Unavailable	( Chen *et al.*, 2016)
Illumina Ribo-Zero rRNA removal kit	HMR	Currently Unavailable	( Pritykin *et al.*, 2017)
Thermo Fisher Scientific RiboMinus™ Eukaryote Kit for RNA-Seq	HMR	Low compatibility with *Drosophila* as stated by the manufacturer	( Lefebvre *et al.*, 2017; Lim *et al.*, 2013; Mugat *et al.*, 2015)
QIAseq FastSelect-rRNA Fly kit	Fly	Inhibits rRNA reverse transcription to cDNA	( de Queiroz *et al.*, 2025; Malwade *et al.*, 2024; Ye *et al.*, 2024)
riboPOOL kit	Fly	Available for both intact and degraded RNA	( Kaneko *et al.*, 2024; van Lopik *et al.*, 2023)

*(HMR – Human, Mouse and Rat)*.

However, due to these reasons mentioned earlier, kits developed for mammals that were used in earlier studies are no longer available or not recommended for use with *Drosophila* samples. However, there are a few promising developments in *Drosophila*-specific rRNA depletion kits like Zymo's Seq RiboFree Total RNA Library Kit, which claims to remove approximately 80% of *Drosophila* rRNA. Also, Qiagen has developed a QIAseq FastSelect–rRNA Fly Kit specifically for *Drosophila* rRNA removal. Similarly, siTOOLs has developed Fly specific riboPOOL kits which use biotinylated probes to deplete rRNA in intact and fragmented RNA samples. Despite the availability and time-saving benefits of commercial kits, they are often inefficient and exorbitantly priced, leading some labs to opt for in-house preparation of rRNA-depletion methods.

There are two primary methods for removing rRNA, each with its own set of advantages and limitations. The first approach, known as pulldown, involves the use of biotinylated DNA oligos that are complementary to rRNA sequences. These oligos hybridize with the rRNA, and the resulting RNA-DNA hybrids are captured using streptavidin-conjugated beads. Once bound, the rRNA can be efficiently removed ( Bhagwat *et al.*, 2014; Chen & Duan, 2011; Culviner *et al.*, 2020). While this method is highly specific and effective for rRNA depletion, it is less suitable for fragmented or degraded RNA samples. The high specificity of the pulldown probes means that if the RNA is fragmented, only portions of the rRNA may bind to the probes, leaving residual rRNA in the sample. Additionally, it requires a large initial amount of starting material, which may not always be feasible. An alternative method is enzymatic depletion, which also relies on the formation of RNA-DNA hybrids ( Baldwin *et al.*, 2021). In this case, the RNase H enzyme is employed to degrade the rRNA specifically bound to the complementary DNA oligos ( Figure 1B). This method has the advantage of being effective even with low-quality or degraded RNA ( Wahl *et al.*, 2022). However, different species require the design of species-specific probes for optimal performance. Both techniques have been used to develop in-house rRNA-depletion kits for *Drosophila*. Biotinylated probes have been developed to remove various rRNA fractions ranging from 2S to 28S rRNA ( Fowler *et al.*, 2018; Thompson *et al.*, 2020). RNase H dependent enzymatic degradation has also been used for the depletion of 5.8S, 18S and 28S rRNA ( Haugen *et al.*, 2024). However, the probe sequences have not been made publicly available.

To address these challenges, we have designed rRNA probes specific for *Drosophila melanogaster* rRNA sequences and demonstrate effective and reproducible depletion of rRNA from adult *Drosophila* brain samples. We make the probe sequences freely available for use by the research community. Our study provides a comprehensive protocol for rRNA-depletion using the RNase H method, from probe design to data analysis, enabling other researchers to replicate and adapt this approach for their studies in *Drosophila melanogaster* and other closely related species.

## Methods

### Fly rearing and genetics

*Drosophila* Canton-S stocks were maintained at 25 ±1°C on cornmeal-sugar-agar media.

### RNA extraction

RNA was extracted from the brains of 5-day-old adult *Drosophila* (N=15) dissected in 1X PBS using TRIzol (Cat No. 15596026, Thermo Fisher Scientific). Briefly, ~15 brains were homogenized in 500 µl of TRIzol until fully dissolved. Subsequently, 100 µl of chloroform was added, and the mixture was vortexed briefly and centrifuged at 13,000g for 10 minutes at 4°C. The aqueous phase was transferred to a fresh tube containing 250 µl of isopropanol to precipitate RNA. The RNA pellet was washed with 70% ethanol and dissolved in 10 µl of nuclease-free water. RNA quality was evaluated using the Agilent Tapestation 4200 before cDNA synthesis, library preparation, and sequencing.

### Probe design and reconstitution

Sequences for 5S (URS00003B4856_7227), 5.8S (URS00005FF212_7227), 18S (URS0000A575BB_7227) and 28S (URS0000A53741_7227) rRNA were sourced from RNAcentral ( Sweeney *et al.*, 2019). Probes were designed using the NEBNext Custom RNA Depletion Design Tool. These probes were analyzed using BLAST to assess potential off-target effects, employing default parameters with specific adjustments for "Organism" ( *Drosophila melanogaster*) and "Optimize for" (blastn) ( Altschul *et al.*, 1990). Subsequently, a final list of probes which covered 98% of rDNA (Table S1) was synthesized by Integrated DNA Technologies, Pvt. Ltd. and reconstituted in nuclease-free water to achieve a stock concentration of 250 µM. These probes were then pooled together, resulting in a final concentration of 2 µM per probe.

### Depletion of rRNA using custom probes

Total RNA which was extracted was split for PolyA enrichment and rRNA depletion. All libraries were prepared using 100 ng of total RNA for poly(A)-selection method and 1 µg of total RNA for rRNA depletion. 2 biological replicates were prepared for PolyA enrichment and rRNA depletion using custom probes. For Poly(A)-enrichment of mRNA, Illumina libraries were prepared using NEBNext Ultra II Directional RNA Library Prep kit (E7765L) and sequenced with Illumina NovaSeq 6000 system. rRNA-depletion was performed as recommended by NEBNext RNA Depletion Core Reagent protocol. Briefly, the RNA integrity was confirmed on an Agilent Tapestation 4200 and samples with at least 7.0 RIN values were used for experiments. The total RNA (500–1000 ng), 2 μL of the 2μM probe pool, 2 μL of the 2μM NEBNext probe hybridization buffer (E6314) and nuclease-free water were mixed to a total volume of 15 μL. This mixture was then incubated at 95°C for 2 min, a touchdown from 95°C to 22°C (0.1°C/sec) and a 5-min hold at 22°C. NEBNext RNA Depletion Core Reagent Set (E7865S) was used for depletion of rRNA where we set up a reaction with 2 μL NEBNext RNase H Reaction Buffer, 2 μL NEBNext Thermostable RNase H and 1 μL nuclease-free water and incubated for 30 min at 50°C. The RNase H treated sample was digested with DNase I and purified using NEBNext RNA Sample Purification Beads. The concentrations of purified RNA samples were measured using the Qubit HS RNA Assay. The purified rRNA-depleted samples were taken for library preparation using NEBNext Ultra™ II Directional RNA Library Prep with Sample Purification Beads (Catalogue no-E7765L) as per the manufacturer's protocol. The libraries were sequenced on the Illumina NovaSeq 6000 platform with a 2x100bp read length. Similarly, rRNA-depletion was performed as recommended by QIAseq FastSelect – rRNA Fly Kits protocol (Cat. No. / ID: 333262) using total RNA extracted from *Drosophila* S2 cells. The rRNA-depleted samples were processed using a similar protocol as described above.

### Data analysis

Sequenced files in FASTQ format were initially assessed for quality using FastQC ( Andrews, 2017/2024). Trimmomatic was used to trim the reads as per the following parameters: *-phred33 HEADCROP:6 LEADING:25 TRAILING:25 AVGQUAL:25 MINLEN:19* ( Bolger *et al.*, 2014). Once high-quality reads were confirmed, alignment was performed against the *Drosophila* dm6 reference genome, using STAR ( Dobin *et al.*, 2013). The count matrix was generated using the featureCounts package ( Liao *et al.*, 2014). Differential expression analysis was conducted using the DESeq2 package in RStudio ( Love *et al.*, 2014). Visualization of results was achieved with EnhancedVolcano for volcano plots and pheatmap for heatmaps ( Kolde, 2010). Picard ( *Broadinstitute/Picard*, 2014/2024) was utilized to calculate gene region coverage, while custom scripts were employed for generating Venn diagrams and bar plots. Ribodetector was used to estimate the rRNA content in each sample ( Deng *et al.*, 2022, p. 2). The softwares used in the study are listed in Table S4.

## Results

### Design and selection of custom probes for *Drosophila* rRNA-depletion

The effectiveness of rRNA removal using RNase H relies on successful hybridization between rRNA and single-stranded DNA probes with sequence homology. Poorly designed probes can impact downstream steps and compromise data quality, making probe design a critical step in rRNA depletion. For this purpose, we downloaded the sequences of *Drosophila melanogaster* 5S (URS00003B4856_7227), 5.8S (URS00005FF212_7227), 18S (URS0000A575BB_7227) and 28S (URS0000A53741_7227) rRNA from RNACentral and designed specific probes using the NEBNext Custom RNA Depletion Design Tool. This process generated 2, 2, 34, and 64 probes for 5S, 5.8S, 18S, and 28S rRNA, respectively. Since RNase H degrades RNA-DNA hybrid regardless of sequence, non-specific probes could inadvertently deplete sequences within our transcriptome of interest. To prevent this, we performed sequence homology searches using BLAST for all the probe sequences, ensuring that non-target RNA with homology to our probes would not be removed unintentionally.

Using the NEBNext Custom RNA Depletion Design Tool, our initial design generated 164 probes, many of which had overlapping sequences. After manual curation of the overlapping probes, we finalized a set of 102 probes (Table S1) that comprehensively covered 98 % of the 5S, 5.8S, 18S, and 28S rRNAs with minimal gaps.

### Custom-generated probes for RNase H based rRNA-depletion performed better than the commercial kit

**Figure 2.**
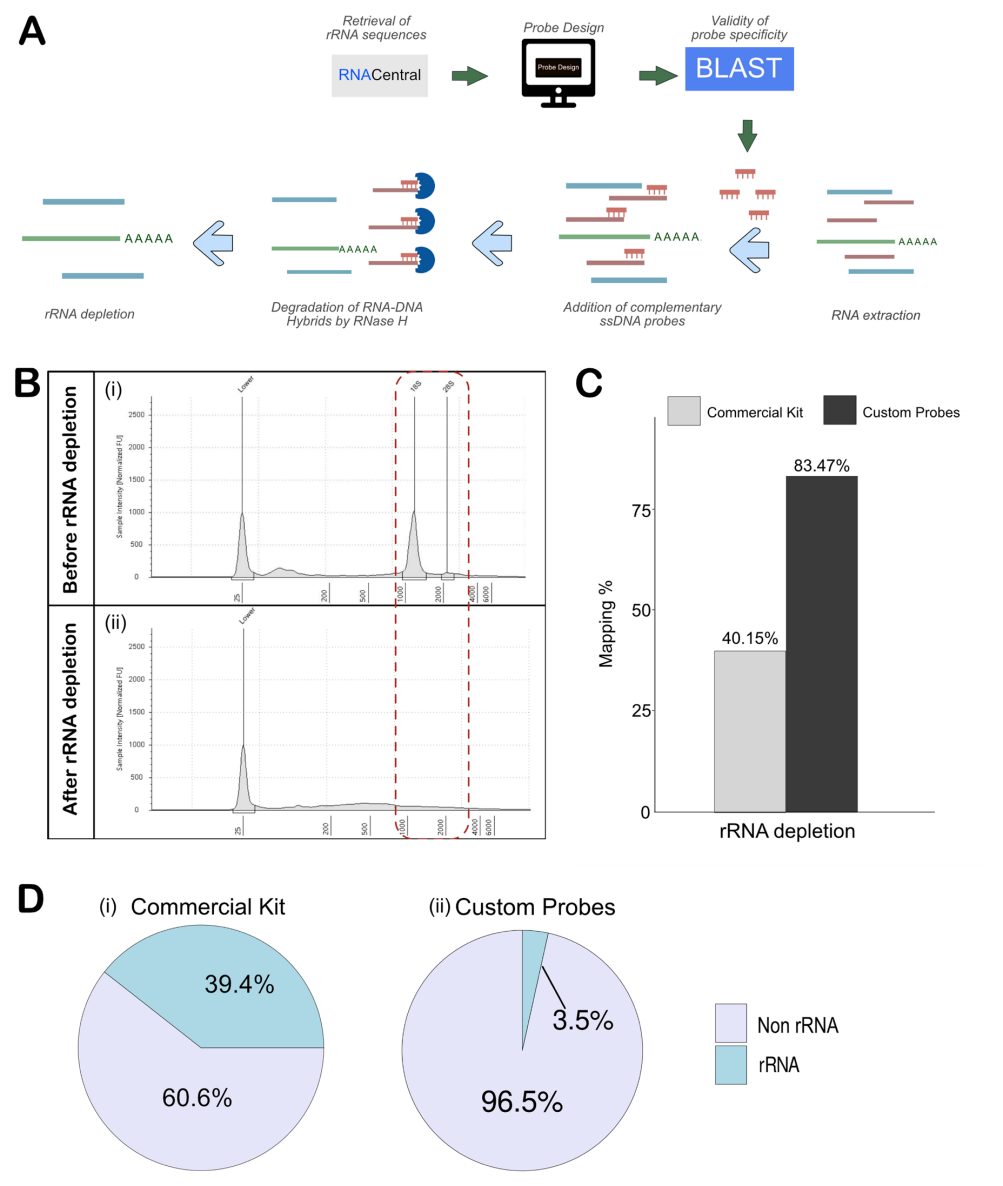
Efficient depletion of rRNA with RNase H using custom-generated probes. ( **A**) Schematic workflow for probe design and rRNA depletion using RNAse H. ( **B**) Tape station analysis shows the reduction in 18S and 28S rRNA after depletion. ( **C**) Mapping percentages of samples sequenced using commercial kit (Qiaseq fast Select-fly rRNA depletion kit) and custom probes (RNAse H Method). ( **D**) Mapped rRNA read percentage from the custom probe and RNAse H method and Qiaseq Fastselect - fly rRNA depletion kit.

RNA was extracted from *Drosophila* brains, and its quality was evaluated using an Agilent Tapestation 4200. The freshly isolated total RNA showed a prominent rRNA peak corresponding to the 18S size ( Figure 2B-i). Unlike mammalian rRNA, which produces two distinct peaks at approximately 1.9 kb and 5 kb for 18S and 28S respectively, *Drosophila* 28S rRNA is naturally fragmented into 28 *S* α and 28S ß which migrates close to the 18S rRNA, resulting in two prominent peaks at approximately 2 kb ( Winnebeck *et al.*, 2010). This total RNA was used for rRNA-depletion using the synthesized custom rRNA probes and RNase H as described in the methods section. The rRNA-depleted samples were evaluated using an Agilent Tapestation 4200, which showed a complete absence of peaks corresponding to 18S and 28S rRNA bands post-depletion ( Figure 2B-ii). An equivalent quality of total RNA extracted from S2 cells was used to deplete rRNA using the QIAseq FastSelect –rRNA Fly Kit. In parallel, we also enriched polyA RNA from both samples to compare the levels of rRNA and ncRNA with respect to rRNA-depleted samples.

Next-generation sequencing (NGS) libraries were generated for rRNA-depleted and polyA-selected RNA using NEBNext Ultra™ II Directional RNA Library Prep Kit. The libraries were sequenced on Illumina NovaSeq 6000, and the reads and quality scores are tabulated in Table S2. FastQC was used to assess the quality of sequenced reads that consistently returned a high quality across all samples, with per-base sequence quality scores exceeding 35.

With the data quality confirmed, the sequencing reads were trimmed as described in the methods section, before mapping to the *Drosophila melanogaster* reference genome (dm6) using STAR. In the samples where custom probes were used for rRNA depletion, the mapping was about 83.47%, which was quite comparable to the polyA-enriched sample. However, the commercial kit-based rRNA depletion removed the rRNA inefficiently, leading to just ~ 40% mapping as compared to the 83.2% mapping in the polyA-enriched sample ( Figure 2C, Table S2). The effectiveness of rRNA removal was determined using Ribodetector, which showed that samples prepared with the QIAseq FastSelect –rRNA Fly kit retained ~39% rRNA, which explains the poor mapping rate ( Figure 2D-i). On the other hand, the custom probe-based method retained just 3.5%, showing efficient rRNA depletion ( Figure 2D-ii). Given the lack of a sufficient number of publications using the QIAseq FastSelect -rRNA Fly kit, it might need optimization. However, our findings indicate that, in its current iteration, the probe-based RNase H method provides superior performance for rRNA depletion.

### Non-coding RNAs were identified in the rRNA-depleted samples

**Figure 3.**
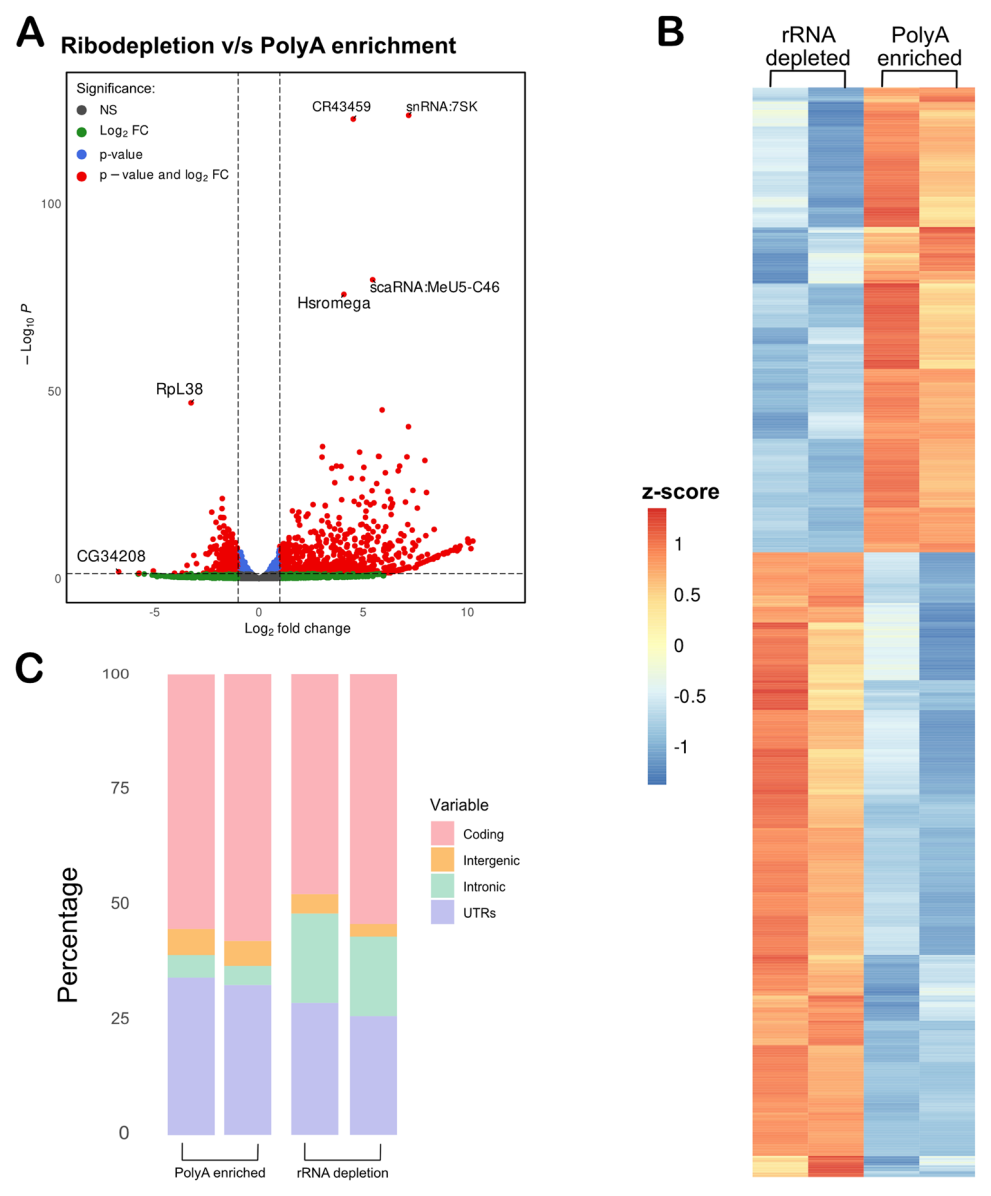
Non-coding RNAs were enriched in the rRNA-depleted samples. ( **A**) A Volcano plot showing RNA targets enriched in rRNA-depleted samples as compared to the polyA-enriched samples. ( **B**) Heat map of > 2-fold change enriched genes with < 0.05 padj value (Fold change values in Table S3). ( **C**) rRNA-depletion increased intronic coverage as compared to the polyA-enriched samples.

The purpose of using rRNA depletion sequencing rather than polyA-selection is to capture a broader population of ncRNAs, which typically lack polyA tail. To evaluate whether the rRNA-depletion method successfully enriched the non-coding transcriptome, we generated count matrices for both rRNA-depleted and polyA-enriched datasets from BAM files using featureCounts. We then performed differential expression analysis by comparing the rRNA count matrix to the polyA count matrix. A volcano plot illustrated the enrichment of RNAs in the rRNA-depleted samples, with several ncRNAs such as CR434459, snRNA:7SK, and hsr-omega prominently upregulated in rRNA-depleted samples ( Figure 3A). A list of these upregulated genes, including their log2 fold changes and adjusted p-values, can be found in Table S3. A heatmap to display the differences in enriched genes between the rRNA-depleted and polyA-selection samples is shown in Figure 3B.

Furthermore, we classified the top 15 ncRNA, ranking them by log2 fold change while excluding those with adjusted p-values greater than 0.05. Most of the identified ncRNA were long non-coding RNAs (lncRNAs), such as CR44157 and CR44474, followed by small nucleolar RNAs (snoRNAs) ( Table 2).

**Table 2.** Top 15 non-coding RNA.

Name	Classification
snoRNA: CG32479-b	sno RNA
snoRNA: Or-aca4	sno RNA
mir-4969	miRNA
mir-2279	miRNA
CR44157	lncRNA
mir-317	miRNA
CR44474	lncRNA
CR43399	lncRNA
CR45256	lncRNA
snoRNA: Psi28S-2622	sno RNA
CR44279	lncRNA
mir-4982	miRNA
CR44496	lncRNA
CR44034	lncRNA
CR44070	lncRNA

In addition to the detection of mRNA and ncRNA, rRNA depletion can capture nascent transcripts, and thus, there is a possibility of higher intronic sequences in rRNA-depletion as compared to polyA-enrichment samples ( Zhao *et al.*, 2018).To confirm this, we used Picard to assess coverage across different genomic regions in both rRNA depleted and polyA samples. Intronic sequences were notably more abundant in the rRNA depleted samples compared to the polyA samples ( Figure 3C). This observation suggests that, while not highly efficient, the rRNA depletion method can detect nascent mRNAs.

Given this, rRNA depletion can be considered a suitable approach for most studies, particularly considering the increasing recognition of the functional importance of ncRNAs. Unless the study specifically requires the detection of low-abundance mRNA targets, rRNA depletion offers a compelling alternative, enabling the exploration of both coding and non-coding RNA dynamics.

## Discussion

Despite being a vital model organism, *Drosophila* has been largely overlooked when it comes to the development of rRNA-depletion kits. Most commercially available kits are specifically optimised for mammalian systems, primarily humans, mice, and rats (HMR). This bias has forced researchers working with *Drosophila* to adapt HMR-optimised kits for rRNA depletion in their studies. However, this approach is suboptimal due to significant differences between *Drosophila* and HMR rRNA structures, particularly the fragmented nature of the 28S rRNA in *Drosophila* compared to the single, continuous 28S rRNA found in mammals. These differences affect the efficiency of rRNA depletion in *Drosophila*, highlighting the need for a species-specific solution.

To fill this gap, we developed a *Drosophila*-specific rRNA depletion method. Although some laboratories have created rRNA depletion techniques for *Drosophila*, their complete probe sequences are unavailable, and their efficiency has not been thoroughly validated or compared to commercial kits ( Haugen *et al.*, 2024; Thompson *et al.*, 2020). Existing commercial kits often rely on biotinylated probes, which are inefficient for fragmented or low-quality RNA ( Culviner *et al.*, 2020). To address these challenges, we designed custom probes specifically targeting *Drosophila* rRNA and paired them with RNase H for efficient depletion. This approach is optimized to enhance efficiency and produces rRNA-depleted RNA suitable for diverse downstream applications, including qRT-PCR, microarray analysis, and NGS.

The NEB Probe Designer tool, used for designing rRNA probes, generates multiple probes with overlapping sequences. To minimize costs and avoid redundancy, these overlapping probes were manually reviewed and eliminated. Due to the short sequence of 2S rRNA (~30 nucleotides), the tool did not design a probe for it. All libraries were prepared using 100 ng of total RNA for poly(A)-selection method and 1 µg of total RNA for rRNA depletion. We did not observe 2S rRNA in our sequencing reads, as it might have been depleted in the RNA purification step following enzymatic digestions. Importantly, the custom probe method produced reproducible results across both replicates.

Further, this method is best suited for the identification of ncRNA in fly tissues. Differential analysis identified several ncRNAs, particularly lncRNAs, were significantly enriched in the rRNA-depleted samples compared to the polyA-enriched ones ( Figure 3A). Previous studies have reported that nascent mRNAs, which typically lack a polyA tail, are not efficiently detected using polyA-enriched samples ( Yang *et al.*, 2011; Zhao *et al.*, 2018). However, rRNA depletion allows nascent RNA detection from the intronic reads ( Figure 3C).

Interestingly, we also detected a few microRNAs (miRNAs) ( Table 2). Given their small size (20–22 nucleotides), miRNAs are typically not expected in rRNA-depleted samples and require specialized sequencing protocols that include specific size-selection steps for enrichment. We hypothesize that these detected miRNAs may belong to a subset of highly abundant ones.

Despite these strengths, a few limitations of the method should be noted. Samples sequenced following rRNA depletion and mapping to the reference genome had a small percentage of reads that were too short to align effectively ("% of reads unmapped: too short"). These could be due to leftover rRNA fragments from depletion. Further STAR documentation does note that “poor sequencing quality, over-trimming, or contamination with ribosomal RNA fragments can yield reads classified as *too short to map*”. Despite this, we obtained mapping percentages of custom probes mediated rRNA depleted samples that were comparable to the polyA selection method ( Figure 2C).

These findings underscore the suitability of rRNA depletion for capturing both coding and non-coding RNA populations, including nascent RNA. Given the high-quality results observed, this method is a robust alternative to commercial kits, and we are confident that this custom probe-based rRNA depletion method will be used more often while sequencing *Drosophila* species as well as closely related insect transcriptomes.

## Ethical and consent statement

The experiments presented in the study were carried out at an appropriate biosafety level laboratory with institute biosafety approvals.

## Data Availability

NCBI Gene Expression Omnibus: RNA Sequencing data from NCBI GEO. Accession ID: GSE282990; http://identifiers.org/geo:GSE282990 ( Koppaka *et al.*, 2024a). The extended data from this study can be accessed from: Figshare: Efficient ribosomal RNA depletion from Drosophila total RNA for next generation sequencing applications. https://doi.org/10.6084/m9.figshare.28093562 ( Koppaka *et al.*, 2024b). The project contains the following extended data: Table S1: List of custom probes used for RNase H mediated rRNA-depletion Table S2: NGS data mapping details Table S3: Differential Expression analysis results Table S4: List of software used in the study Data are available under the terms of the Creative Commons Attribution 4.0 International license (CC-BY 4.0).
