# Supplementary material for: EFFICIENT RIBOSOMAL RNA DEPLETION FROM DROSOPHILA TOTAL RNA FOR NEXT-GENERATION SEQUENCING APPLICATIONS
Source: Wellcome Open Res. 2025 May 13;10:112. Originally published 2025 Feb 28. [Version 2] doi: 10.12688/wellcomeopenres.23509.2 (PMC12123297; doi:10.12688/wellcomeopenres.23509.2)
Supplement: Peer review history

# Reviewer report: Reviewer response for version 2

**DOI:** 10.21956/wellcomeopenres.26694.r123472

**Published:** 2025-05-29

**Authors:** Jean-Yves Roignant

**Affiliations:** 1 University of Lausanne, Lausanne, Switzerland

I went through author's responses and I am satisfied with the revisions. I therefore approve the article without reservation.

Is the rationale for developing the new method (or application) clearly explained?

Yes

Is the description of the method technically sound?

Yes

Are the conclusions about the method and its performance adequately supported by the findings presented in the article?

Partly

If any results are presented, are all the source data underlying the results available to ensure full reproducibility?

Yes

Are sufficient details provided to allow replication of the method development and its use by others?

Yes

Reviewer Expertise:

NA

I confirm that I have read this submission and believe that I have an appropriate level of expertise to confirm that it is of an acceptable scientific standard.

# Reviewer report: Reviewer response for version 2

**DOI:** 10.21956/wellcomeopenres.26694.r123471

**Published:** 2025-05-27

**Authors:** Amaresh Chandra Panda

**Affiliations:** 1 Institute of Life Sciences, Bhubaneswar, Odisha, India

The manuscript has improved significantly after the revision. The authors have answered/revised the manuscript according to the suggestions. I have no further comments to make.

Is the rationale for developing the new method (or application) clearly explained?

Yes

Is the description of the method technically sound?

Partly

Are the conclusions about the method and its performance adequately supported by the findings presented in the article?

Partly

If any results are presented, are all the source data underlying the results available to ensure full reproducibility?

Yes

Are sufficient details provided to allow replication of the method development and its use by others?

Yes

Reviewer Expertise:

RNA sequencing, rRNA depletion, non-coding RNA

I confirm that I have read this submission and believe that I have an appropriate level of expertise to confirm that it is of an acceptable scientific standard.

# Reviewer report: Reviewer response for version 1

**DOI:** 10.21956/wellcomeopenres.25929.r121320

**Published:** 2025-04-16

**Authors:** Jean-Yves Roignant

**Affiliations:** 1 University of Lausanne, Lausanne, Switzerland

In this manuscript the authors develop a method to deplete ribosomal RNA in Drosophila melanogaster. They claim that current kits are not optimized for Drosophila, and therefore new methods should be implemented.

 I have not doubt that this would be a great addition for the drosophila community. Nevertheless the authors omitted to mention the kits from siTOOLs that were specifically developed for Drosophila, one for full RNA and another for degraded RNA. They should definitively mention these kits in TABLE1 and ideally compare these kits with their method.

 The authors used Ribodetector, a new tool developed for removing rRNA reads which was trained in the human genome. The authors could check the rRNA reads using bowtie with the *Drosophila* genome to check if they would get similar results.

 The authors mentioned the difference in mapped reads between the probe system and the kit system, it would be interesting to know the type of unmapped reads. Is it related to the difference of number of rRNA reads? It would be interesting to know if the number of mapped reads would change if the step of removing rRNA reads was performed before or after the mapping with STAR. Since normally, rRNA reads are removed before mapping but the authors perform it after mapping with STAR.

Is the rationale for developing the new method (or application) clearly explained?

Yes

Is the description of the method technically sound?

Yes

Are the conclusions about the method and its performance adequately supported by the findings presented in the article?

Partly

If any results are presented, are all the source data underlying the results available to ensure full reproducibility?

Yes

Are sufficient details provided to allow replication of the method development and its use by others?

Yes

Reviewer Expertise:

Drosophila, molecular biology

I confirm that I have read this submission and believe that I have an appropriate level of expertise to confirm that it is of an acceptable scientific standard, however I have significant reservations, as outlined above.

# Response

**Published:** 2025-05-01

**Authors:** Baskar Bakthavachalu

**Affiliations:** School of Biosciences and Bioengineering, Indian Institute of Technology Mandi, Mandi, Himachal Pradesh, India

We thank the reviewers for their thorough evaluation of our manuscript and the constructive feedback. The suggestions have been instrumental in improving the clarity of our data presentation and strengthening the rigor of the methods section. Below, we provide detailed responses to each of the reviewers’ comments.

***1. Nevertheless, the authors omitted to mention the kits from siTOOLs that were specifically developed for Drosophila, one for full RNA and another for degraded RNA. They should definitively mention these kits in TABLE1 and ideally compare these kits with their method.*** We thank the reviewer for pointing this out and have now included the siTOOLs in the revised manuscript and in Table-1. However, a direct comparison will not be possible, as our study is focused specifically on depleting rRNA using custom probes, rather than conducting a comparative analysis of different methods.

***2. The authors used Ribodetector, a new tool developed for removing rRNA reads which was trained in the human genome. The authors could check the rRNA reads using bowtie with the Drosophila genome to check if they would get similar results.*** While we acknowledge the reviewer's concern regarding the use of Ribodetector for estimating Drosophila rRNA reads, it is important to note that Ribodetector relies on the human genome solely for the estimation of false positives and false negatives. The tool was trained using sequences from the SILVA database, which does not specifically focus on human rRNA sequences. In fact, Ribodetector has been widely used for rRNA estimation in non-human genomes as well (Dyksma & Pester, 2023; Nenciarini et al., 2024; Zhang et al., 2024). To further address the reviewer's query, we constructed additional analysis using 1000 randomly selected 100 bp reads from the fly rRNA sequences and another 1000 sequences from the whole dm6 reference genome. Our analysis showed that Ribodetector was able to correctly identify 98.6% of rRNA sequences while detecting only one rRNA sequence out of a thousand from the dm6 genome suggesting the tool is compatible for use with Drosophila rRNA.

***3. The authors mentioned the difference in mapped reads between the probe system and the kit system, it would be interesting to know the type of unmapped reads. Is it related to the difference of number of rRNA reads? It would be interesting to know if the number of mapped reads would change if the step of removing rRNA reads was performed before or after the mapping with STAR. Since normally, rRNA reads are removed before mapping but the authors perform it after mapping with STAR.*** Unmapped reads typically originate from various sources. During our quality assessment of sequencing data generated from probe-based rRNA depletion, using FASTQC, we observed duplication rates ranging between 7–12%. These duplicates are primarily introduced during library preparation and PCR amplification and are subsequently removed post-alignment using Picard Tools. Notably, multimapping rates exceeding 15% often indicate inadequate rRNA depletion; our samples remained below this threshold. Therefore, a multimapping rate of 7–12% is considered acceptable for libraries prepared using custom rRNA depletion probes (Dobin & Gingeras, 2015; Fu et al., 2018; Picard Tools - Broad Institute). Approximately 5–6% of the reads were classified as "too short," which could result from sample contamination or residual rRNA. However, given that poly(A)-selected libraries also show a similar proportion (~5%) of short reads, this fraction in our rRNA-depleted libraries is not considered significant. Additionally, we observed a small fraction (~0.4%) of reads that failed to align due to factors other than mismatches or short length, an expected and acceptable rate (Dobin & Gingeras, 2015). As recommended by the reviewer, we aligned reads from the custom probe-based rRNA-depleted libraries to rRNA sequences, extracted the unmapped reads, and subsequently aligned them to the Drosophila melanogaster dm6 reference genome. This process resulted in only a marginal improvement in mapping efficiency, which is consistent with the low levels of rRNA contamination detected by Ribodetector. We applied the same approach to sequencing data from the kit-based rRNA depletion method where we notice ~7% of the reads maps to multiple loci, while ~57% of the reads are too short for mapping, suggesting potential issues at the step of rRNA depletion or cDNA synthesis. While we agree that a deeper characterization of the unmapped reads could be informative, we consider this beyond the scope of the current study. Nonetheless, those interested in further analysis can do so using the publicly available raw data under accession number GSE282990.

# Reviewer report: Reviewer response for version 1

**DOI:** 10.21956/wellcomeopenres.25929.r119970

**Published:** 2025-03-27

**Authors:** Amaresh Chandra Panda

**Affiliations:** 1 Institute of Life Sciences, Bhubaneswar, Odisha, India

In this manuscript, Koppaka & colleagues developed a cost-effective rRNA-depletion method for Drosophila. Here, they designed DNA probes against the rRNA and optimized the efficient digestion of the rRNA using RNase H. The authors claim that this method lead to better depletion of rRNA and enrichment of noncoding RNA in the drosophila RNA-seq library.

 Although this is a fascinating study, I have a few comments to further improve the work.

1. The number of RNA-seq reads in commercial kit samples is ~20% of the reads obtained in the custom probe sample. The higher percentage of rRNA reads and the low detection of non-coding RNAs in the commercial kit could be attributed to the sequencing depth. Similarly, in the polyA library, the read numbers are also very low compared to custom probe library. It might be possible that the buffer used for rRNA depletion by the commercial kit may be incompatible/inefficient for the cDNA library preparation by the library kit. Also, if there are any differences in the amount of libraries made from equal amounts of starting RNA, they may be discussed. 
2. The major conclusions of the article are based on a single library prepared with custom probes compared to a library prepared from a commercial kit. In addition, there is a huge difference in the depth of sequencing as shown by the million reads.  It would be better to use at least two biological replicates with similar sequencing depth in RNA-seq to increase confidence in the differential expression analysis, as well as the differences observed in rRNA depletion efficiency.
3. It might be better to mention the % of rRNA sequence covered by the probes. It might be better to discuss the fate of the portions of rRNA that are not targeted by the probes after digestion.
4. The commercial kit used for comparison uses rRNA depletion by pulldown while the method used here used RNase H mediated digestion. Using a kit that use RNase H for rRNA depletion might have been a better comparison.
5.  Table 2 mentions one poly (A) library with a custom probe. It is unclear whether the polyA selection was performed after rRNA depletion using a custom probe or a commercial kit for rRNA depletion. Please revise the methods section for a better understanding of samples and their comparisons. It might be better to include the sample table in the main text/Figure.
6. The tapestation data for before and after rRNA depletion using a commercial kit may be provided.

Is the rationale for developing the new method (or application) clearly explained?

Yes

Is the description of the method technically sound?

Partly

Are the conclusions about the method and its performance adequately supported by the findings presented in the article?

Partly

If any results are presented, are all the source data underlying the results available to ensure full reproducibility?

Yes

Are sufficient details provided to allow replication of the method development and its use by others?

Yes

Reviewer Expertise:

RNA sequencing, rRNA depletion, non-coding RNA

I confirm that I have read this submission and believe that I have an appropriate level of expertise to confirm that it is of an acceptable scientific standard, however I have significant reservations, as outlined above.

# Response

**Published:** 2025-05-01

**Authors:** Baskar Bakthavachalu

**Affiliations:** School of Biosciences and Bioengineering, Indian Institute of Technology Mandi, Mandi, Himachal Pradesh, India

We thank the reviewer for his careful evaluation of our manuscript and for the constructive feedback provided. The comments have been valuable in enhancing the clarity of our data presentation and the rigor of the methods section. Below, we address each of the reviewers’ queries in detail.

***1. The number of RNA-seq reads in commercial kit samples is ~20% of the reads obtained in the custom probe sample. The higher percentage of rRNA reads and the low detection of non-coding RNAs in the commercial kit could be attributed to the sequencing depth. Similarly, in the polyA library, the read numbers are also very low compared to custom probe library. It might be possible that the buffer used for rRNA depletion by the commercial kit may be incompatible/inefficient for the cDNA library preparation by the library kit. Also, if there are any differences in the number of libraries made from equal amounts of starting RNA, they may be discussed.***

**Response: **This study was designed to demonstrate an effective rRNA depletion method. The method was compared with a kit that was available with us. Normally, a sequencing depth of around 10 million is sufficient to perform differential expression analysis (Liu et al., 2014). The Drosophila melanogaster transcriptome is roughly 60Mb and a total bases needed for 20X coverage will be about 1.2 Gb. So, about 6 million paired-end reads of 200 bp will be sufficient to identify most transcripts. Both our custom probe-based method as well as kit-based rRNA depleted samples have reads beyond this estimated depth. We obtained 25 million paired-end reads for kit based rRNA depletion, while about 11 million paired-end reads for the same sample that was enriched using PolyA beads which is well within the acceptable range. In addition, in our original submission, we seemed to have accidentally mentioned just the single end read depth data, but in actual, we had used the double that read depth which is now corrected in the supplementary data Table 2 of the revised submission. It is also available under accession number GSE282990. QIAseq® FastSelect™ Handbook has a section that describes cDNA synthesis and library preparation using NEBNext Ultra II Directional Library Prep Kit and so it is unlikely that the reagents of the rRNA depletion kit is incompatible with cDNA library preparation. That said, though we obtained a relatively lower number of reads for PolyA-enriched samples, we found better mapping percentages as compared to rRNA depletion, suggesting this could not be the reason for the poor performance of the kit.  All libraries were prepared using 100 ng of total RNA for poly(A)-selection method and 1 µg of total RNA for rRNA depletion. The custom probe method produced reproducible results across both replicates, and this has now been included in the methods and discussion section.

***2. The major conclusions of the article are based on a single library prepared with custom probes compared to a library prepared from a commercial kit. In addition, there is a huge difference in the depth of sequencing as shown by the million reads.  It would be better to use at least two biological replicates with similar sequencing depth in RNA-seq to increase confidence in the differential expression analysis, as well as the differences observed in rRNA depletion efficiency.***

**Response: **We agree with the reviewer that at least two biological replicates are essential for the custom probe to be an acceptable method of choice for rRNA depletion. We had actually performed the experiments with two biological replicates. The data from both replicates are comparable, as shown in the revised supplementary table 2 and available under accession number GSE282990. The confusion regarding the use of a single library may have arisen because we initially reported the average sequencing depth across samples. We have now corrected this, and the updated table reflects the sequencing depth of each replicate individually. Furthermore, differential expression analysis was conducted between the two replicates of the PolyA-enriched samples and the two replicates of the rRNA-depleted samples, each with equivalent sequencing depth.

***3. It might be better to mention the % of rRNA sequence covered by the probes. It might be better to discuss the fate of the portions of rRNA that are not targeted by the probes after digestion.***

**Response: **Approximately 98% of the rRNA sequences (5S, 5.8S, 18S, and 28S) were covered by our probes. This has been explicitly mentioned in the revised manuscript. The regions not covered by the probes were extremely short. They would be eliminated during bead-based RNA cleanup following the RNAse H treatment.

***4. The commercial kit used for comparison uses rRNA depletion by pulldown while the method used here used RNase H mediated digestion. Using a kit that uses RNase H for rRNA depletion might have been a better comparison.***

**Response: **The Qiagen FastSelect Fly rRNA depletion kit does not employ a pulldown-based method. Instead, it uses oligos to inhibit reverse transcription into cDNA, thereby preventing rRNA sequences from being incorporated into the final sequencing library. To our knowledge, there is currently no commercially available rRNA depletion kit that utilizes an RNase H-based method for rRNA depletion in Drosophila. Finally, we would like to emphasize that the primary objective of this study is to demonstrate the efficiency of the custom probes in depleting rRNA, rather than to conduct a comparative analysis against existing commercial kits.

***5. Table 2 mentions one poly (A) library with a custom probe. It is unclear whether the polyA selection was performed after rRNA depletion using a custom probe or a commercial kit for rRNA depletion. Please revise the methods section for a better understanding of samples and their comparisons. It might be better to include the sample table in the main text/Figure.***

**Response: **In our method, total RNA was extracted and subsequently divided for PolyA enrichment and rRNA depletion. Two biological replicates were prepared for each condition: PolyA-enriched samples and rRNA-depleted samples using custom probes. We have now revised Table 2 and the methods section to more accurately reflect these steps.

***6. The tapestation data for before and after rRNA depletion using a commercial kit may be provided.***

**Response: **In RNA sequencing workflows, RNA is typically converted into cDNA, which is then used for sequencing. The Qiagen FastSelect Fly rRNA kit specifically inhibits reverse transcription of rRNA. Consequently, while rRNA remains physically present in the reaction mixture, it does not advance to later stages of library preparation. As a result, rRNA can still be detected by quality control systems such as the Tapestation, even when depletion has been effective resulting in visualization of peaks corresponding to rRNA bands. Thus, the efficacy of rRNA depletion cannot be accurately assessed based on Tapestation results. This is also explicitly mentioned in page 12 of QIAseq® FastSelect™ Handbook - “It is not possible to test the efficiency of the FastSelect reaction by running a portion of the eluate from the bead cleanup on a Bioanalyzer®, TapeStation®, Fragment Analyzer™, etc. FastSelect works by inhibiting reverse transcription of rRNA, which does not occur until the first-strand synthesis reaction during library prep.”
